# In Vitro Regeneration of *Capparis spinosa* L. by Using a Temporary Immersion System

**DOI:** 10.3390/plants8060177

**Published:** 2019-06-16

**Authors:** Valeria Gianguzzi, Paolo Inglese, Ettore Barone, Francesco Sottile

**Affiliations:** 1 Department of Agricultural, Food and Forest Sciences (SAAF), University of Palermo, 90128 Palermo, Italy; valeria.gianguzzi@unipa.it (V.G.); paolo.inglese@unipa.it (P.I.); ettore.barone@unipa.it (E.B.); 2 Department of Architecture (DARCH), University of Palermo, 90128 Palermo, Italy

**Keywords:** *Capparis spinosa*, PlantForm bioreactor, micropropagation, temporary immersion system (TIS)

## Abstract

Three caper (*Capparis spinosa* L.) biotypes grown on the Sicilian island of Salina (38°33′49″ N) were micropropagated to evaluate two different in vitro culture systems: one using the traditional solid medium, and the other based on liquid culture in a PlantForm bioreactor. PlantForm is a temporary immersion system (TIS), a new propagation method in which the shoots undergo temporary immersion in a liquid medium in order to avoid the accumulation of gas through forced ventilation. This study proposes a protocol to improve the efficiency of in vitro propagation of caper plants, while also reducing production costs, because of the elimination of the gelling agent, and manual labor, requiring limited subcultures and posing minimal contamination risks. Our results show that the caper shoots propagated in bioreactors demonstrated good adaptability and better growth rates than those grown in the conventional system. Statistically significant differences were observed between plants grown in the PlantForm liquid culture and those grown in solid medium regarding the number and length of shoots, which were further promoted by the addition of plant growth regulators (PGRs). The relative growth and real proliferation rate of the caper explants were higher when using meta-Topolin than when using 6-benzylaminopurine as a PGR. Overall, the TIS improved in vitro caper culture by promoting the proliferation, length, and vigor of the shoots.

## 1. Introduction

The genus *Capparis* belongs to the family Capparaceae which includes about 250 species [1]. The caper (*Capparis spinosa* L.) is a perennial xerophytic shrub naturally spread throughout the Mediterranean basin, with two subspecies: *C. spinosa* subsp. *spinosa* and *C. spinosa* subsp. *rupestris* [2], but also widely diffused both in the wild and in the cultivated form. The most important area for its commercial production is Southern Europe (Italy, Spain, and Greece), where it is cultivated both in traditional and in specialized systems (especially for its flower buds, but also for its unripe fruits and young shoots, which are pickled in salt or vinegar and used as a condiment) [3]. More recently, caper cultivation has been the object of significant commercial interest in several areas, particularly in North Africa (Tunisia, Morocco, and Egypt) and in the Middle East (Syria), according to its different uses. In Italy, caper crops are mainly distributed in the south, and traditional production is concentrated in the small islands around Sicily (mostly, Pantelleria and the Aeolian Islands) where the species plays an important economic role [4,5]. The importance of caper production is linked to its use in the food industry as well as in medicine and cosmetics [6,7,8]. From an agronomic point of view, there are several aspects that must be carefully evaluated in order to improve caper yield and quality. One of the most important aspects is related to the propagation system. Most studies of caper propagation concern methods for increasing the seed germination rate, which is usually low due to seed dormancy [9] and the high degree of seed heterozygosity [10]. Therefore, in order to meet farmers’ needs to increase caper production, vegetative methods are now preferred for the propagation of selected caper biotypes. However, the main problems related to vegetative propagation by stem cuttings appear to be dependent on the type of propagation material, affected by the environment [11]. It is well known that even in cultivation areas where the shoots are able to reach a consistent caliber, lignify well, and accumulate an adequate amount of reserves, the percentage of rooting rarely exceeds 50%. For these reason, in recent years there has been an increasing interest in micropropagation, with the aim of rapidly obtaining genetically homogenous and uniform plant material suitable for more specialized plantings. On the other hand, somaclonal variations are often observed in micropropagated plants, probably deriving from the massive use of hormones in the production cycle [12]. In vitro caper culture was reported for the first time in 1984 [13]; soon after, it was reported by several authors, which all mainly used nodal segments as the starting material [14,15,16,17,18,19,20,21,22]. In vitro growth and development are determined by plant growth regulators (PGRs) which are responsible for cell division and growth. PGRs (auxin and cytokinin) regulate the development of different plant organs during in vitro grown. Auxins (IAA, IBA, NAA, and 2,4-D) induce cell elongation and tissue swelling, cell division (callus proliferation), and the formation of adventitious roots; cytokinins (kinetin, BAP, mT, 2ip, and ZEA) stimulate growth and development; they also improve cellular division and induce the formation of adventitious shoots by decreasing the apical dominance [23]. Only a few studies are available on caper in vitro propagation and they are mainly focused on the initial phases of propagation starting from vegetative material [18,19,24]. These studies have shown that the best results, in terms of induction of axillary growth, were obtained when cytokinins (mainly BAP) and auxins were used either alone or in combination.

Notwithstanding the positive results in terms of multiplication, especially with respect to in vivo propagation, in vitro culture has not made it possible to develop efficient protocols from a nursery point of view, and this has led to the search of alternative solutions able to combine high multiplication rates and reduced production costs through the use of innovative techniques based on temporary immersion systems (TIS).

TIS technology, via a bioreactor, is a mechanized system used in plant tissue culture to improve large-scale micropropagation in laboratories and research centers [25,26,27]. The bioreactor was technologically developed as an alternative to the conventional micropropagation process, which aims to optimize in vitro cultivation through process automation. These mechanized systems ensure greater contact between explant and culture medium, allowing a greater number of plants to propagate in less time and space. Furthermore, compared with systems using a solid medium, TIS allow a greater absorption of nutrients by the explant and greater aeration in the in vitro environment. [28]. The usefulness of TIS and their positive effects in one or more stages of plant tissue proliferation have been reported for several easily multiplying species such as *Pinus radiata* [26]; banana [27]; *Saccharum* spp. [29]; *Ananas comosus* [30]; *Pseudotsuga menziesii*; *Pinus taeda* [31]; and *Psidium guaiava* [32]. In TIS, the immersion times vary greatly, probably because of the large variety of species, micropropagation processes, and types of temporary immersion system used. De Rezende Maciel et al. [33] showed that long immersion times (1 h every 6 h) were efficient for potatoes, whereas very short immersion times (1 min every 12 h) stimulated somatic embryo production in *Coffea arabica* and rubber and increased grapevine shoot propagation [34]. Nevertheless, to the best of our knowledge, there are no available studies concerning the application of TIS techniques on recalcitrant species such as the caper, whereas few papers have reported data on forestry trees [35,36].

Therefore, the aim of this study was to develop high-efficiency micropropagation protocols using the TIS proposed by PlantForm [35,36,37,38].

## 2. Materials and Methods

The plant material was obtained from 12-year-old plants of Sicilian biotypes of *C. spinosa* L. grown on the Aeolian island of Salina (38°33′49″ N). Three accessions were considered in the present study: “Sal 39”, “Sal 37”, and “Sal 35”, following preliminary observations carried out in the same cultural environment by the Department of Agricultural, Food, and Forestry Sciences of the University of Palermo, aimed at determining the agronomic value of several cultivated accessions. “Sal 39”, “Sal 37”, and “Sal 35” have been providing an abundant caper production that has remained constant over the years in a sufficiently compact harvest period. Furthermore, they have proven to be able to produce standard high-quality flower buds of excellent firmness, even after traditional processing, both in salting and in brine systems (data not published). In early June, apical shoots were collected directly in the plots where the three accessions were selected. After removing the leaves, the samples were then washed in running water and cut into nodal segments (1 cm in length). The nodal explants were sterilized by immersion in a 70% ethanol solution for 5 min and then in a 2% sodium hypochlorite solution for 20 min followed by three 5 min rinses in sterile distilled water. After rinsing, the base of the nodal segments was slightly trimmed in order to avoid any damage caused by the permanence of sodium hypochlorite.

### 2.1. Culture Media and Conditions

#### 2.1.1. Solid Medium

**Figure 1:**
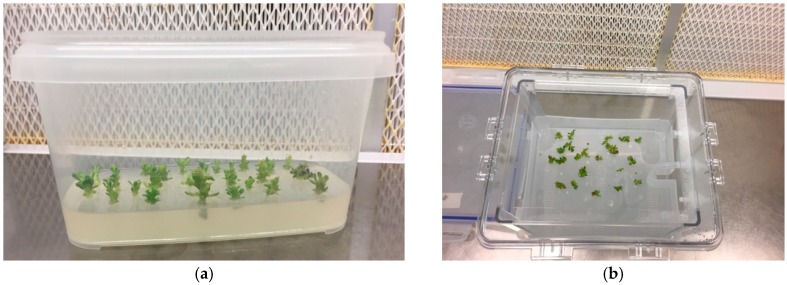
Placement of “Sal 39” explants to start the multiplication phase in the bioreactor (**a**) and on traditional solid medium (**b**).

Once sterilized, the explants were raised in Microbox vessels containing MS basal medium including vitamins [39,40], supplemented with 30 g/L sucrose and 8 g/L Plant agar (Duchefa) as a gelling agent, were put at 25 ± 1 °C in the dark, and then placed under a cool white fluorescent lamp, with a photosynthetic photon flux density (PPFD) of 35 μmol m^−1^ s^−1^ and a photoperiod of 16 h, for 30 or 60 days without any subculturing. In order to compare the effects of different growth regulators on the formation of axillary shoots, two cytokinins were tested, i.e., 6-benzylaminopurine (BAP) and meta-Topolin (mT) in both media at 6.6 µM concentration, whereas indole-3-butyric acid (IBA) at 0.25 µM concentration was used as an auxin source. After 4 weeks, the explants obtained from the introduction phase in vitro were used for the multiplication phase (Figure 1). Nodal segments (1 cm, on average) with multiple axillary buds were isolated from the shoots and subcultured on MS including vitamin substrates integrated with:

- Substrate A1: 6.6 µM BAP (Sigma-Aldrich B-3408), 0.25 µM IBA (Sigma-Aldrich I-5.386);
- Substrate A2: 6.6 µM mT (Duchefa T0941), 0.25 µM IBA.

#### 2.1.2. PlantForm Temporary Immersion System

In this study, the temporary immersion cycle was controlled by an electronic system set up as follows: two dives per day for two minutes and two air changes per day for two minutes. Air injection was performed using 0.20 μ-pore filters to promote sterilization [41]. Preliminarily, all parts of the bioreactor were sterilized in an autoclave for 20 min at 120 °C. The culture substrates used were the same as those on a solid substrate; each bioreactor contained 500 mL of medium. 

In our experiments, the effects of the different cultivation methods on the multiplication were determined at 30 and 60 days in the following way:

Relative growth rate (RGR) was calculated on a fresh-weight basis using the following equation:

RGR = [(*Wf − Wi*)/(*tf* − *ti*)] * 100

where *ti* is the starting day of the experiment, *tf* is the final day of the experiment (final day of subculture), *Wi* is the weight of the initial biomass (at *ti*), *Wf* is the final biomass weight (at *tf*), and *tf* − *ti* indicates the number of days of subculture;

Absolute growth rate (AGR) of shoots was calculated as follows:

AGR = final microcuttings/initial microcuttings

The RGR is the instantaneous rate of growth relative to the culture biomass and, therefore, it is an index of the immediate response to treatment, since it measures the proportional growth rate per unit of time; the AGR is a parameter that characterizes the total real growth of the explants at the end of treatment. Both are influenced by the genotype, the PGRs present in the substrate (quantity and quality), and the culture method adopted. 

The number and the length of the shoots were also measured in all experiments.

### 2.2. Data Analysis 

Each treatment included 25 explants. Five replications (Microbox vessel) and five replication explants were submitted to each trial for growth in solid medium. In the TIS, 25 explants were placed inside each bioreactor. Statistical analysis was performed using SYSTAT 13. To highlight statistically significant differences and possible interactions between culture system and PGRs, the two-way analysis of variance (ANOVA) was performed (*p* ≤ 0.05). One-way ANOVA was performed when the interaction between two factors was not significant; each factor was analyzed individually, and the separation of the averages was performed via the Tukey test (*p* ≤ 0.05).

## 3. Results and Discussion

**Figure 2:**
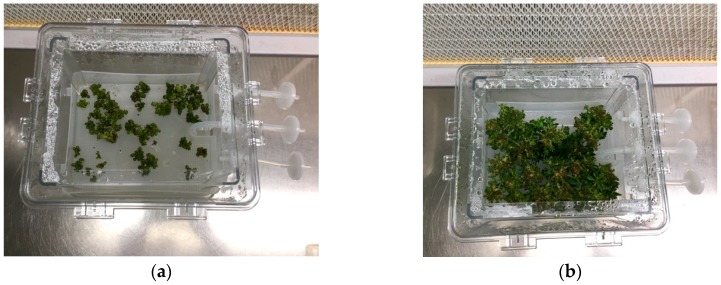
Explants of caper accessions “Sal 37” (**a**) and “Sal 39” (**b**) in a bioreactor after 60 days.

**Figure 3:**
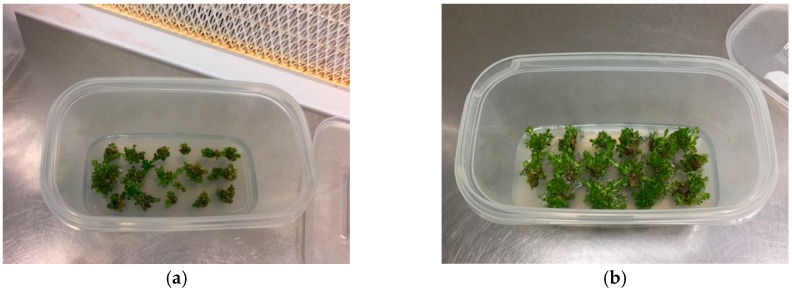
Explants of caper accessions “Sal 37” (**a**) and “Sal 39” (**b**) in solid substrate after 60 days.

The results obtained showed that the three selected caper accessions responded differently to the in vitro culture systems and culture media adopted. Both factors—culture system and PGR concentration—positively affected the proliferation of *C. spinosa* L. Significant differences were observed between culture systems after 30 and 60 days. Overall, the *C. spinosa* shoots produced in PlantForm showed good adaptability and a better growth rate than those grown on a solid substrate (*p* ≤ 0.05). The highest RGR value was recorded when mT was used as a source of cytokinin to replace BAP. In all comparisons between the two culture systems adopted, temporary immersion in a twin-tube (liquid) gave the best results (Figure 2 and Figure 3).

**Figure 4:**
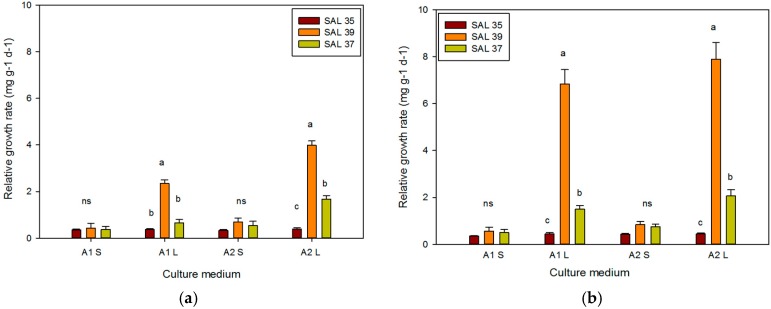
Relative Growth Rate (RGR) of different *Capparis spinosa* accessions after 30 (**a**) and 60 days (**b**) in different culture systems. Bars represent standard error. Abbreviations: A1 S: substrate A1 solid, A1 L: substrate A1 liquid; A2 S: substrate A2 solid; A2 L: substrate A2 liquid. The different letters reported above the bars grouped for each single treatment indicate statistically significant differences; n.s. not significant (Tukey’s test, *p* ≤ 0.05).

The selection “Sal 39” showed the best RGR results (3.98 mg g^−1^ d^−1^) in the substrate containing mT after 30 days in the TIS; on the other hand, with the solid medium, an RGR equal to 0.69 mg g^−1^ d^−1^ was obtained. On the contrary, the “Sal 35” selection showed a reduced adaption to all of the different culture systems, showing the lowest RGR (Figure 4). The RGR, calculated at 30 and 60 days, showed an improvement of culture growth in the TIS compared to the solid system. Similar results were obtained for *Olea europaea* L. cv Canino [42], using the same PlantForm facilities, for which an increasing RGR was observed depending on the hormone concentration used in the medium. In the present study, a high RGR value was recorded even after 60 days of continuous culture (without subculture). In fact, the RGR in the TIS shoot culture containing 6.6 μM mT + 0.25 μM IBA was about 10 times higher than the one in the solid medium (7.89 versus 0.84 mg g^−1^ d^−1^).

**Figure 5:**
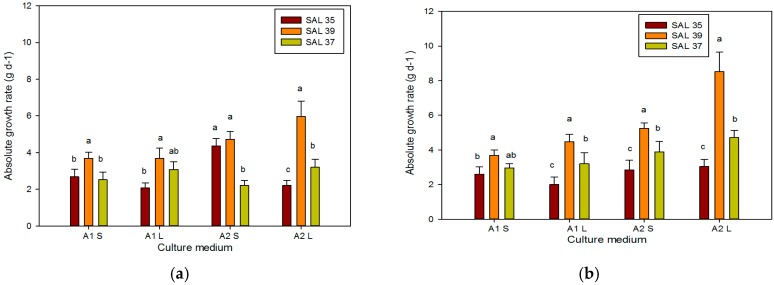
Absolute growth rate (AGR) of different *C. spinosa* accessions after 30 (**a**) and 60 days (**b**) in different culture systems. Bars represent standard error. The different letters reported above the bars grouped for each single treatment indicate statistically significant differences; n.s. not significant (Tukey’s test, *p* ≤ 0.05).

Regarding the analysis of the AGR, significantly different results were obtained using mT. The best results in terms of AGR were obtained for “Sal 39” in liquid medium containing mT at both 30 and 60 days of subculture (5.96 and 8.52 g d^−1^, respectively). Again, the “Sal 35” selection showed the lowest AGR among the tested accessions (Figure 5).

**Figure 6:**
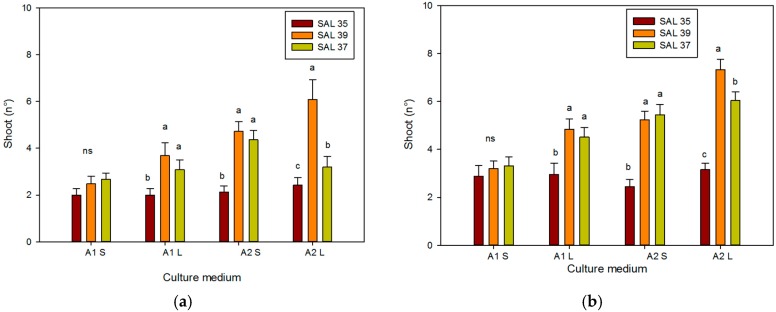
Effect of different culture systems and plant growth regulator (PGR) concentrations on the number of shoots in different *C. spinosa* accessions after 30 (**a**) and 60 days (**b**). Bars represent standard error. The different letters reported above the bars grouped for each single treatment indicate statistically significant differences; n.s. not significant (Tukey’s test, *p* ≤ 0.05).

The statistical analysis performed on growth parameters showed a significant interaction (*p* ≤ 0.05) between the culture medium and the PGRs adopted, affecting the length and the number of shoots. A higher number of shoots was obtained in the TIS compared to the solid medium (7.32 vs 5.24 on average, respectively) in the presence of mT (Figure 6). The induction of axillary buds was greater on MS integrated with 6.6 μM mT + 0.25 μM IBA compared to the substrate containing BAP.

**Figure 7:**
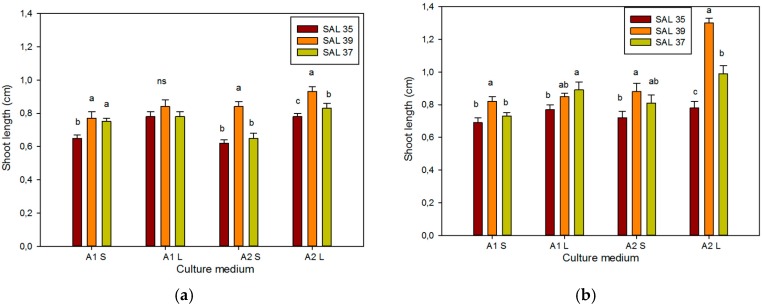
Effect of different culture systems and PGR concentrations on shoot length in different *C. spinosa* accessions after 30 (**a**) and 60 days (**b**) of culture. Bars represent standard error. The different letters reported above the bars grouped for each single treatment indicate statistically significant differences; n.s. not significant (Tukey’s test, *p* ≤ 0.05).

**Figure 8:**
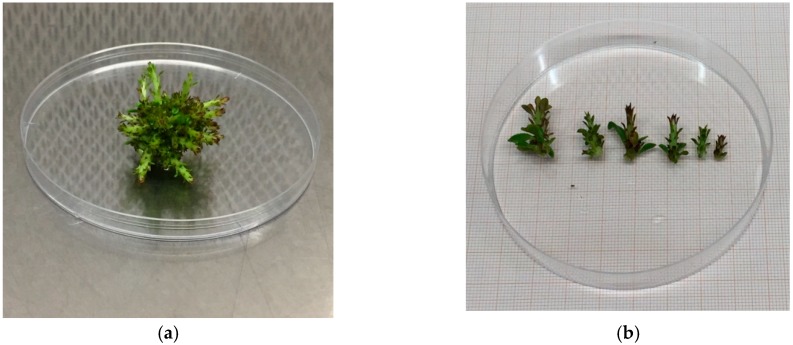
“Sal 39” explant obtained in a bioreactor after 60 days (**a**) and detail of the length of each shoot (**b**).

**Figure 9:**
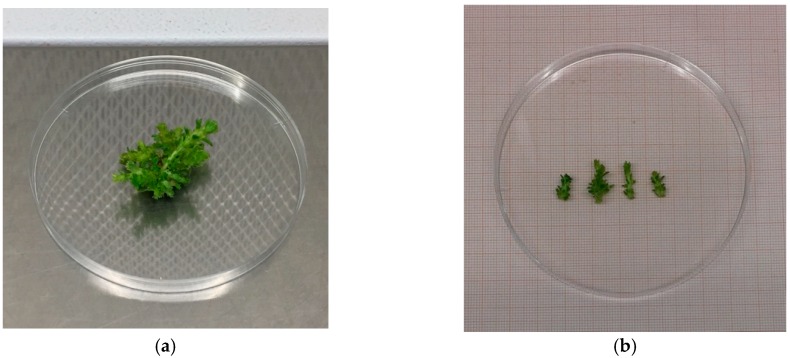
“Sal 39” explant obtained in a solid medium after 60 days (**a**) and detail of the length of each shoot (**b**).

In previous studies [20] using solid MS with 6 μM BAP + 0.12 μM IBA for multiplication, 8.9 ± 1.6 shoots for explant were obtained, whereas, by placing explants in solid MS supplemented with 88 mM of sucrose and 6.6 μM BAP + 0.25 μM IBA, 8 to 10 shoots were generated; finally, 4 shoots per explants were obtained in a solid MS medium with 4 μM of BAP [17]. In the present study, for “Sal 39”, after 60 days of culture, in the substrate containing BAP, it was possible to obtain between 3.2 shoots, measuring 0.82 cm in length in solid medium, and 4.84 shoots, measuring 0.95 cm in length in liquid medium, while for “Sal 37” an average of 3.32 shoots were obtained in solid substrate, and up to 4.52 in liquid substrate. For “Sal 39”, in the medium containing mT, an average of 5.24 shoots with a length of 0.95 cm were obtained in the solid medium, and up to 7.32 shoots with a length of 1.3 cm were obtained in the liquid medium. “Sal 37” produced 5.44 shoots in the solid medium and up to 6.04 in the liquid medium. A greater explant length was obtained after 60 days in the TIS, compared to the solid medium (1.3 cm vs 0.95 cm) for “Sal 39”, using MS medium integrated with mT (Figure 7). The other two selections “Sal 37” and “Sal 35” had shorter shoots. The data obtained showed the role played by both PGRs and culture system in elongation tissue growth (Figure 8 and Figure 9).

Many studies have shown that BAP has a significant effect on the multiplication of shoots. For example, BAP has proven to be an efficient proliferating agent in all assayed concentrations by stimulating the simultaneous growth of multiple caper shoots [17]. Furthermore, another study concerning in vitro conservation of *Cadaba eterotricha* (Capparaceae) showed that different concentrations of BAP in MS medium had a significant effect on the frequency of shoot regeneration (65%) and on the number of shoots per explant [43]. Similar results were reported in a recent study about micropropagation of *C. spinosa* L. [19]. In the present study, the best results were obtained in the MS medium integrated with meta-Topolin. The efficiency of mT has also been reported for a number of plant species different from caper [44,45,46,47].

## 4. Conclusions

This study aimed to understand the usefulness of PlantForm bioreactors as a valid alternative to traditional solid-substrate techniques, in terms of proliferation indexes, relative growth rate, and number and length of shoots. It should be noted that no previous study with TIS techniques has ever been performed on this species, which is specifically defined as “recalcitrant” to agamic propagation. Nonetheless, the adoption of a TIS has been reported for other species with generalized positive effects in one or more stages of proliferation of plant tissues. The results obtained in the present study allow us to highlight the comparative value of TIS and solid medium during the multiplication phase of three different caper selected accessions cultivated in the Aeolian island of Salina. As a whole, we observed a higher RGR in TIS compared to the solid medium system, together with better proliferation indices and higher number of shoots produced by the explants, in accordance with previous results obtained for other plant species [46,47,48,49]. Bioreactor cultures have several advantages compared to those on agar substrate, such as a better control of plant tissue contact with the culture medium, optimal supply of nutrients and growth regulators, as well as better aeration and substrate circulation, filtration of the medium, and reduction of the number of cultures. In addition, the TIS can improve the propagation protocol by introducing a semi-automatic cultivation process, forced ventilation, and liquid medium, which promote more effective nutrient absorption. In general, it can be concluded that an increase in mass production can be obtained through the use of liquid medium, even in caper cultures. The availability of mineral nutrients depends on the type of crop, agar-gelled or liquid substrate, and type and size of the explants. As a consequence, the duration and frequency of TIS immersion are parameters of paramount importance that must be carefully considered for micropropagation success. This aspect can partly influence the size of the shoots, for example, by reducing their length and number. It should, however, be noted that, as generally reported in plant tissues culture, the genotype effect is quite relevant, as confirmed in this present study. Our data, in fact, showed that some biotypes are more adaptable to in vitro multiplication techniques, confirming that it is not easy to develop a single protocol for each species and, thus, it is necessary to test the adaptation of each genotype.
